# Potential Application of Algae in Biodegradation of Phenol: A Review and Bibliometric Study

**DOI:** 10.3390/plants10122677

**Published:** 2021-12-06

**Authors:** Syahirah Batrisyia Mohamed Radziff, Siti Aqlima Ahmad, Noor Azmi Shaharuddin, Faradina Merican, Yih-Yih Kok, Azham Zulkharnain, Claudio Gomez-Fuentes, Chiew-Yen Wong

**Affiliations:** 1 Department of Biochemistry, Faculty of Biotechnology and Biomolecular Sciences, Universiti Putra Malaysia, Serdang 43400, Selangor, Malaysia; syahirahbatrisyia@gmail.com (S.B.M.R.); aqlima@upm.edu.my (S.A.A.); noorazmi@upm.edu.my (N.A.S.); 2 Center for Research and Antarctic Environmental Monitoring (CIMAA), Universidad de Magallanes, Avda. Bulnes, Punta Arenas 01855, Chile; claudio.gomez@umag.cl; 3 School of Biological Sciences, Universiti Sains Malaysia, Minden, Gelugor 11800, Penang, Malaysia; faradina@usm.my; 4 Division of Applied Biomedical Sciences and Biotechnology, School of Health Sciences, International Medical University, Bukit Jalil, Kuala Lumpur 57000, Selangor, Malaysia; yihyih_kok@imu.edu.my; 5 Department of Bioscience and Engineering, College of Systems Engineering and Science, Shibaura Institute of Technology, Saitama-shi 337-8570, Saitama, Japan; azham@shibaura-it.ac.jp; 6 Department of Chemical Engineering, Universidad de Magallanes, Avda. Bulnes, Punta Arenas 01855, Chile

**Keywords:** phenol, phenolic compounds, biodegradation, phycoremediation, algae, hazardous pollutant

## Abstract

One of the most severe environmental issues affecting the sustainable growth of human society is water pollution. Phenolic compounds are toxic, hazardous and carcinogenic to humans and animals even at low concentrations. Thus, it is compulsory to remove the compounds from polluted wastewater before being discharged into the ecosystem. Biotechnology has been coping with environmental problems using a broad spectrum of microorganisms and biocatalysts to establish innovative techniques for biodegradation. Biological treatment is preferable as it is cost-effective in removing organic pollutants, including phenol. The advantages and the enzymes involved in the metabolic degradation of phenol render the efficiency of microalgae in the degradation process. The focus of this review is to explore the trends in publication (within the year of 2000–2020) through bibliometric analysis and the mechanisms involved in algae phenol degradation. Current studies and publications on the use of algae in bioremediation have been observed to expand due to environmental problems and the versatility of microalgae. VOSviewer and SciMAT software were used in this review to further analyse the links and interaction of the selected keywords. It was noted that publication is advancing, with China, Spain and the United States dominating the studies with total publications of 36, 28 and 22, respectively. Hence, this review will provide an insight into the trends and potential use of algae in degradation.

## 1. Introduction

In recent years, the increase in the global transportation of hazardous chemicals has led to accidental spillage of chemicals into the environment. Phenol is a common chemical associated with accidental spillage [1] and is widespread as an environmental contaminant. Besides, phenol is a toxic compound listed as a priority pollutant by the United States Environmental Protection Agency. The enlistment is because of the acute and chronic toxicity of the compounds to humans and animals [2]. 

The increase of industrialisation and overexploitation of natural resources has also affected the environment [3,4]. The treatment of water contaminated with phenolic pollutants is challenging as the compounds exist in different concentrations from various industrial processes. Wastewater with phenolic compounds leads to severe damage owing to its low biodegradability and high solubility in water [5,6]. Hence, numerous wastewater treatment techniques have been developed to remove phenolic compounds from domestic, industrial and municipal wastewater. Developing the techniques is imperative to reduce the destructive impact of phenol on humans and aquatic animals. The rise of water pollution leads to sustainable approaches to restore the environment from phenolic pollutants. Recently, more emphasis has been put on environmentally friendly approaches to overcome the rising water pollution problem and the imbalance of the aquatic ecosystem [7,8]. 

Phycoremediation is a technique used for treating chemically contaminated water using algae [9]. This technique also ensures no transportation of toxic compounds to the treatment sites via adsorption by the algae [10]. Phycoremediation technique is now successfully replacing physiochemical methods in the remediation of the environment due to the unique characteristics of algae in assimilating various toxic pollutants in aromatic hydrocarbon, phenols, heavy metal and organochlorine [11,12]. Algae have been effectively used for wastewater treatment owing to their intrinsic property for removing nutrient, metal and organic compounds [13,14]. Besides, algae could utilise phenol as a single carbon source [15,16,17]. At present, algae from the genus *Chlorella*, *Spirulina*, *Scenedesmus* and *Chlamydomonas* are the notable non-pathogenic representatives of microalgae that have been employed in phycoremediation of phenolic compounds [18]. Ubiquitous distribution and production of in situ oxygen are desirable factors for algae in wastewater treatment [19,20,21]. Interestingly, algae can be used for the long-term protection of the environment from toxic compounds. This review will cover topic pertaining to mechanisms involved in phenol degradation by algae.

## 2. Bibliometric Analysis

The term bibliometric was first coined by Alan Pritchard in 1969 and has been widely employed in recent years [22,23,24,25]. Bibliometric analysis is a quantitative method that amalgamates mathematical and statistical analyses. Besides, this analysis reveals hot trends in research and uncover the researchers’ publications, collaborations between institutions, and academic quality [26,27]. 

**Figure 1:**
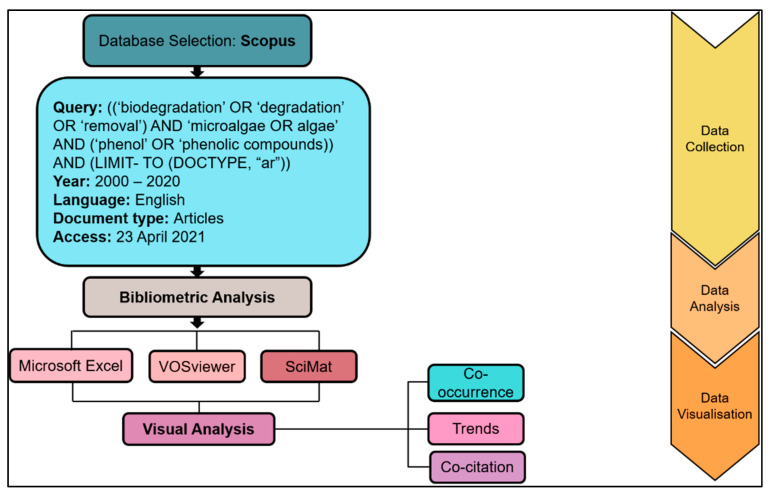
The workflow of the bibliometric analysis.

This review focuses on identifying trends in related fields and exploring potential paths for further research using microalgae-based bioremediation, especially in phenol degradation. To accomplish this, the available literature was mapped using a bibliometric technique to assess and analyse the issues that drawn the most interest from the scientific researchers and their advancement. An appropriate bibliometric analysis is indispensable to distinguish and assess the evolution and dynamics of the research field. Microsoft Excel, VOSviewer software (version 1.6.16, Center for Science and Technology Studies, Leiden University, The Netherlands), and SciMAT were used to analyse the topic that piqued the curiosity of the scientific community (Figure 1). The bibliometric analysis was done on publications from the year 2000–2020. Scopus databases were used for the extraction of data. Scopus is a user-friendly search interface that grants access to a broad spectrum of scientific databases and citations [28]. However, the access provided by Elsevier requires an access fee. Figure 1 shows the general flow in retrieving the information about the research topic. Comprehensive data extraction and analysis of scientific publications for the literature review are vital in establishing and solving co-current research. A gap can be easily identified in this manner. This quantitative method primarily involves evaluating research in numerous disciplines by ranking publications based on authors, journal sources and institutions.

### 2.1. Trends in Publication

**Figure 2:**
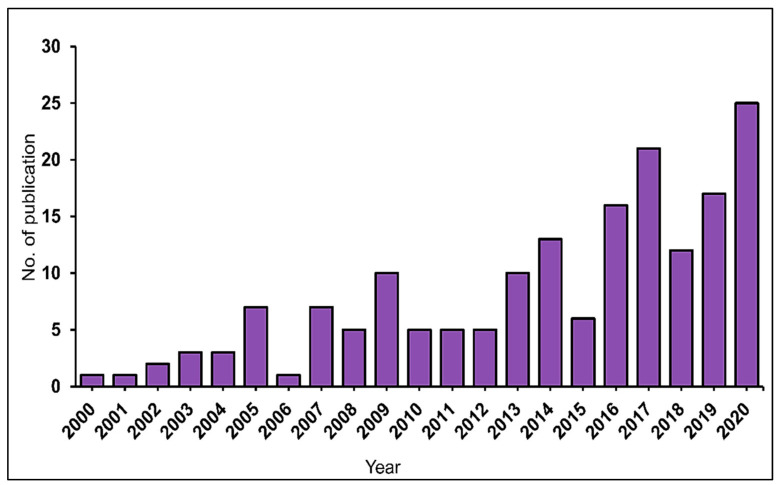
Distribution of publication from 2000–2020.

A fluctuating trend in the number of articles published per year can be observed in Figure 2. Less than 10 articles were published annually during the first period of assessment (2000–2005), and a slow increase in publication was recorded during the third period (2010–2015). Conversely, the fourth period (2016–2020) had remarkable growth, with the average number of publications being more significant than the cumulative number of articles before 2015. Therefore, this demonstrates that using microalgae in remediating phenol pollutants has been gaining the attention of researchers, and the increment is likely to continue.

### 2.2. Analysis Based on Subject Areas

**Figure 3:**
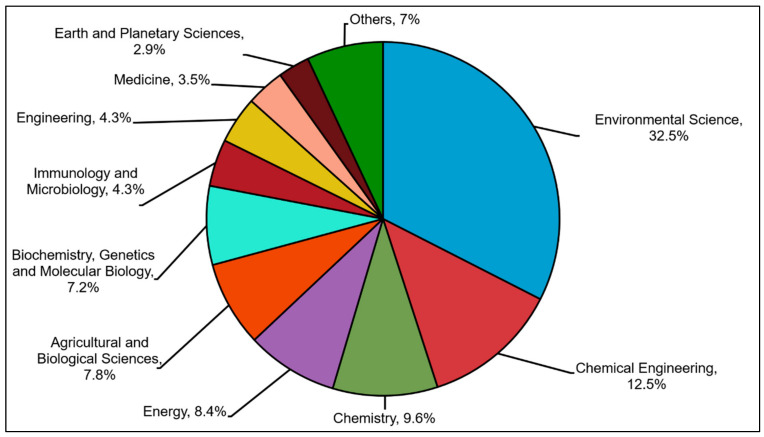
Distribution publication on subject areas.

Figure 3 displays the distribution of the central theme of this review, in which the environmental sciences possessed the highest percentage (32.5%), followed by chemical engineering (12.5%). This distribution can also show the hot trends in the research topic and explore the selected topics by a scientist with different fields of study. Hence, environmental sciences pay particular attention, especially in the remediation of phenol using microalgae.

### 2.3. Countries with the Highest Work Published

**Figure 4:**
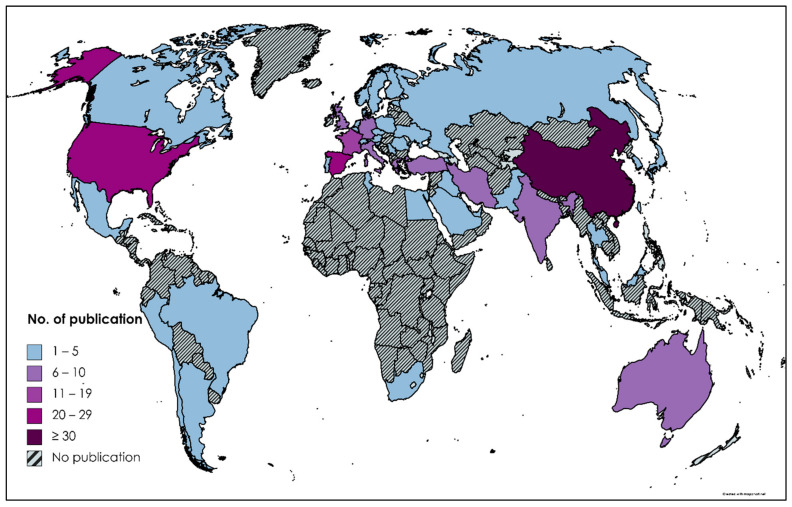
Global representation of the number of the publication. The map was created with mapchart (https://mapchart.net/) accessed date 13 August 2021.

The publication number provides insight for researchers to identify the global trends and increase collaboration in their respective fields of study. China, Spain, United States, France, Germany, India, Italy, Turkey, Australia and Greece were the top ten countries contributing the most to the research topic. The highest number of countries per region came from Europe (24), Asia (15), North America (3), Africa (3), South America (3) and Oceania (1). The higher the publications, the darker the shade (Figure 4). Three countries: China, Spain and the United States, gained the spotlight with contributions of 38, 26 and 22 publications, respectively. Undeniably, developing countries are dominating the research as they have greater concern for the sustainability of remediation. China is the largest remediation market globally, and the domination in this research was influenced by the greater understanding of the polluted sites and active commitment to managing contaminated sites [29].

## 3. Analysis Using SciMAT

SciMat is a powerful visualisation tool designated based on the mapping analysis approach and accustomed to the main themes’ evolution [30]. Interestingly, this open-source software offers diverse analysis and visualisation outcomes in such cluster networks, strategic diagrams, evolution maps, and overlapping. 

For the analysis using SciMAT, the time interval of the year 2000–2020 was separated into four distinct time periods which are 2000–2005, 2006–2010, 2011–2015 and 2016–2020. By doing so, ensuring that each of the periods had a comparable quantity of articles. 

### 3.1. Strategic Diagram

**Figure 5:**
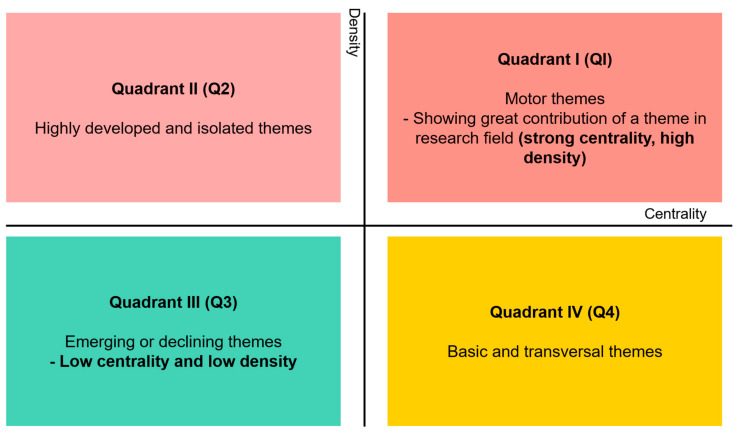
The strategic diagram (outcome) from SciMAT as described by Cobo et al. [30].

SciMAT visualisation also includes a strategic diagram. The cartesian plane is shown in this strategic diagram. The centrality is denoted on the x-axis, while the density of related keywords on the y-axis allows the evaluation of research studies. The density relates to the internal strength of the network, whereas the centrality shows the connection between a network with other networks [31]. In addition, the node size corresponds with the number of the publication. Four quadrants are represented in the strategic diagram where each quadrant gives a different interpretation (Figure 5).

#### 3.1.1. First Period (2000–2005)

**Figure 6:**
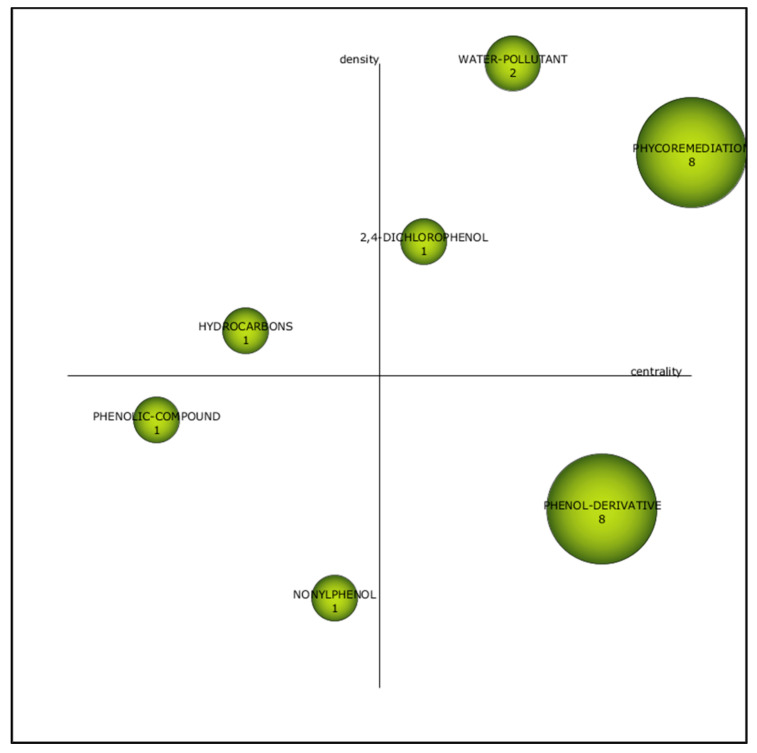
Strategic diagram for the first period (2000–2005).

Seven main themes were identified from documents concerning phenol degradation (Figure 6). “Phycoremediation”, “2,4-dichlorophenol” and “water pollutant” were the motor themes during the first period. “Phycoremediation” emerged as the most developed motor theme with strong centrality (0.50), and eight documents associated with this theme. Phycoremediation has been discovered as a novel technology in recent years. The employment of bacteria is the most prevalent bioremediation approach, and it is now primarily viewed as conventional bioremediation technology. Phycoremediation is a technique that uses photosynthetic algae to biologically transform waste into harmless compounds [9,10]. This approach has emerged as a possible alternative for pollutants segregation. This shows that algae can be associated with the removal of contaminants in such as heavy metals and aromatic compounds [32]. The promising characteristics of algae further enhance their use in the removal of pollutants compared to higher aquatic. 

Despite being a cluster with low development, “phenol derivative” was observed as significant due to its high centrality (0.83) in the basic theme (Table 1). Hence, “phenol derivative” would be a promising theme in the research study.

#### 3.1.2. Second Period (2006–2010)

**Figure 7:**
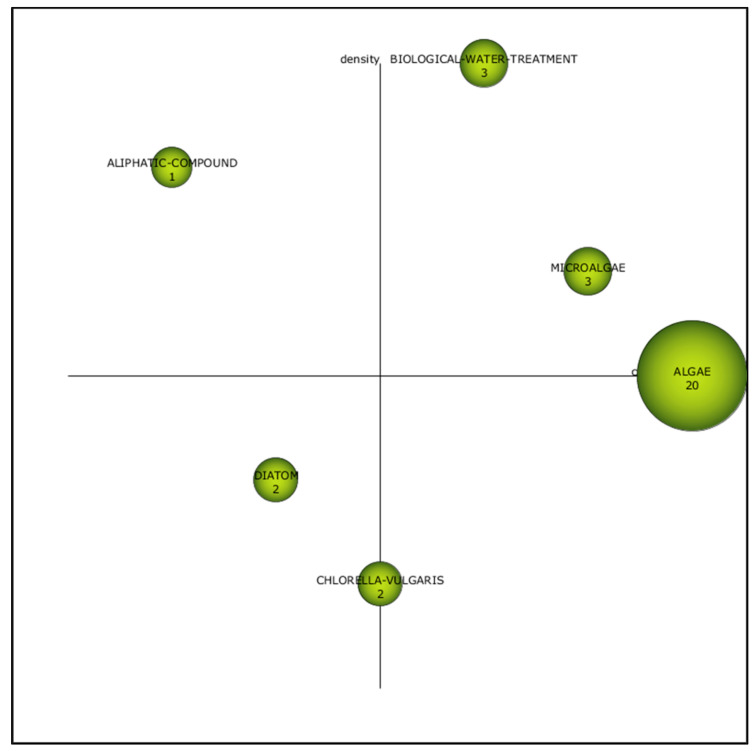
Strategic diagram for the second period (2006–2010).

In the second period of (2006–2010), “biological water treatment” and “microalgae” were the motor themes (Figure 7) with centrality value of 0.67 and 0.83, respectively (Table 2). It is critical to engage in appropriate treatment strategies to counteract the escalating environmental issues. The treatment method employed shall ensure the eradication of phenol to a permissible discharge limit. The concentration and volume of the treated effluent and cost of treatment should be considered when choosing the best methods. 

The removal of phenolic contaminants can be done either through biological or physiochemical treatment. The physiochemical treatment of phenol includes adsorption [33], ion exchange [34], electro Fenton method [35,36,37], oxidation [38], membrane filtration [39,40], flocculation and coagulation process [41,42]. Adsorption is one of the physiochemical approaches focusing on treating wastewater polluted with dyes, heavy metals and organic and inorganic pollutants [43]. Adsorption is a well-studied treatment approach due to the phenol affinity to the active surface of carbon [44]. Due to the high cost of operation using activated carbon, the material used in this method is typically obtained from low-cost agricultural waste [45]. Hence, this absorbent is used to remove and recover wastewater streams from phenolic pollutants efficiently. 

Chemical oxidation is another physiochemical approach that uses chemical agents to convert toxic contaminants to less harmful compounds [46]. This alternative is favourable when wastewater is flooded with high contaminant concentration, since it uses a strong oxidant as the chemical agent. Hydrogen peroxide is a commonly used oxidant for initiating oxidation reactions [47].

**Figure 8:**
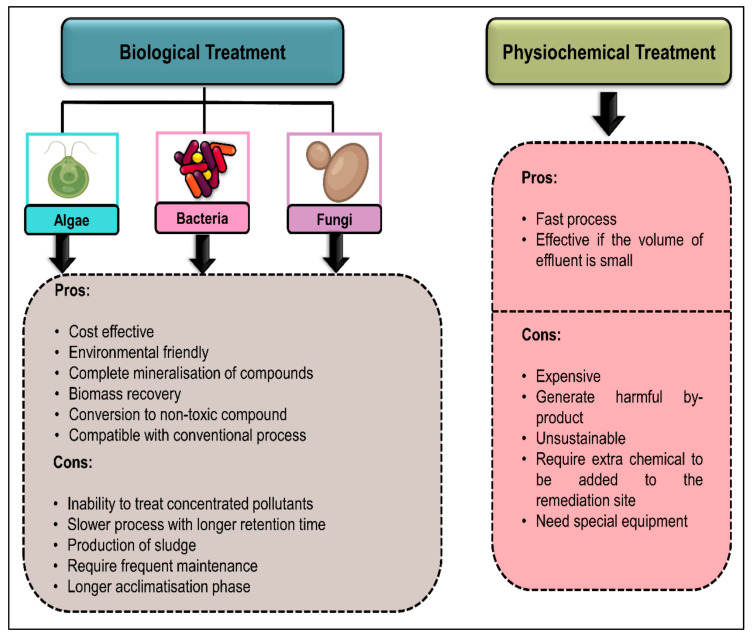
The comparison between biological and physiochemical treatment as described by [10,21,51,54].

Biological treatment employs microorganisms, or the enzymes secreted by a specific microorganism and transforms the wastes into simple end products [48,49,50]. The demands of biological treatments rise as it is a promising approach in removing organic pollutants, including phenol [51,52,53]. Biological treatment is still regarded as an attractive and structured alternative for the removal of phenol as it confers more advantages than physiochemical treatment (Figure 8). 

Meanwhile, themes related to “diatom” and “*Chlorella vulgaris*” were still emerging, making it possible to initiate future research exploration (Figure 7). “Aliphatic compound” was the most developed theme with the centrality of 0.17 (Table 2). This theme has a close internal link but an infirm external link. This means that the theme is not too influential in this research field. Although it is not the central attention in phenol degradation, it is a stable topic in this field of study.

Figure 7 also highlights that “algae” is the basic and transversal theme. This cluster theme was illustrated as a theme with low density (0.50) and high centrality value (1); hence, the themes possess greater impact yet slower evolution in the research field (Table 2). The theme was also characterised as a theme with a weak internal link with other topics. However, it is still crucial in the phenol degradation topic.

#### 3.1.3. Third Period (2011–2015)

**Figure 9:**
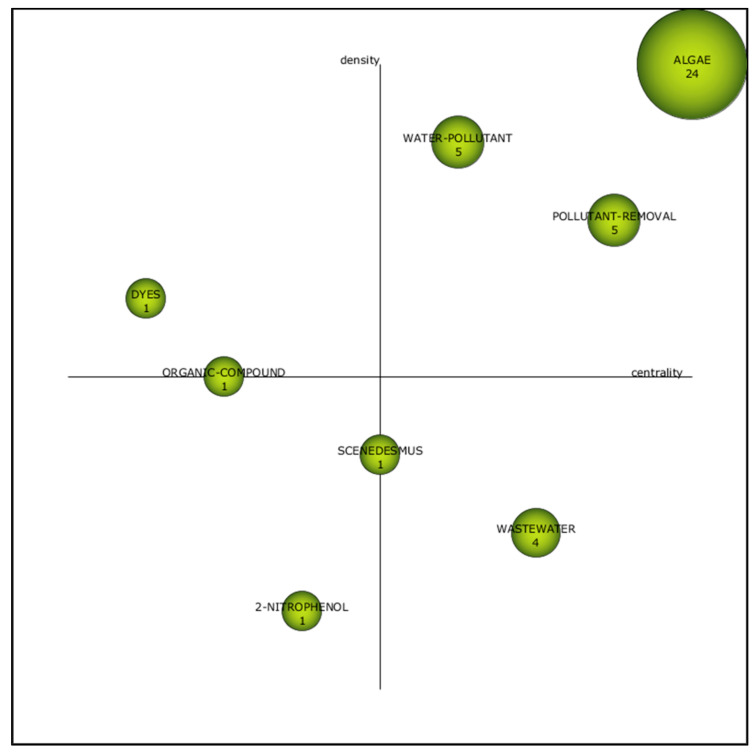
Strategic diagram of the third period (2011–2015).

The motor themes for this the third period of (2011–2015) were “algae”, “pollutant removal” and “water pollutant” (Figure 9). These topics were essential in phenol removal studies since they have higher density and strong centrality value (1,1), (0.75,0.88) and (0.88,0.62), respectively (Table 3). The term “algae” has shifted from the fourth quadrant (2006–2010) (Figure 7) to the first quadrant during this period (Figure 9), with higher number of documents (24). The study on “2-nitrophenol” and “*Scenedesmus*” was not receiving the attention of the research group during this period. Both themes fall at the declining theme quadrant, with the low centrality value of 0.38 and 0.50, respectively. 

During the third period, the developed themes included “organic compound” and “dyes” with the centrality of 0.25 and 0.12, respectively (Table 3). The h-index for “algae” was the highest (18) (Table 3), showing that this topic has been receiving special attention and vast application in phenol degradation. It is worth noting that the term “water pollutant” remained as the motor theme during the first period (2000–2005) (Figure 6) and this period (Figure 9). This proves that the theme receiving research attention and influence with regards to the phenol removal studies.

#### 3.1.4. Fourth Period (2016–2020)

**Figure 10:**
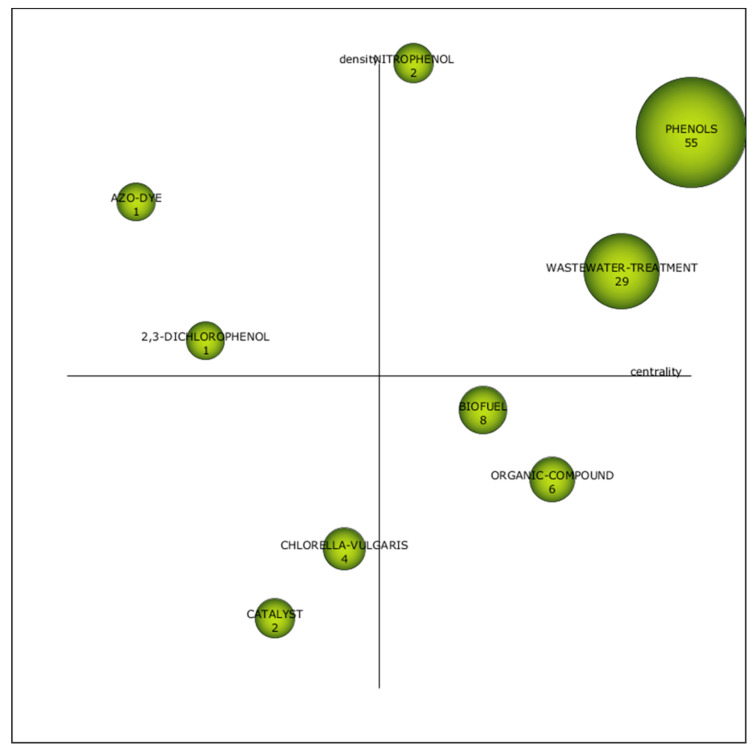
Schematic diagram for the fourth period (2016–2020).

There are three motor themes, two isolated, two emergent and two basic themes, as shown in Figure 10. The term “phenols” associated with 55 documents was the highly dense and central cluster, indicating influential research and a close internal relationship. “*Chlorella vulgaris*” and “catalyst” were the emerging themes for this period, with centrality values of 0.44 and 0.33, respectively (Table 4). These topics were not the central research attention based on their position in an immature quadrant. Figure 10 also highlights that “biofuel” and “organic compound” are the basic and transversal themes. The term “organic compounds” shifted from most developed theme during the third period (2011–2015) (Figure 9) to basic theme in this period; however, with enhancement of documents numbers (6).

### 3.2. Thematic Network- The Central Cluster of Each Period

Thematic networks supplement the strategic diagram by illustrating how each of the strategic diagram’s theme is related to any other themes in the [30,55]. Thematic networks will enhance the understanding of the association between phenols and other issues throughout time. Therefore, a theme that gives precedence to those with high impact application was chosen, since there are several themes in the strategic diagram.

#### 3.2.1. First Period (2000–2005)

**Figure 11:**
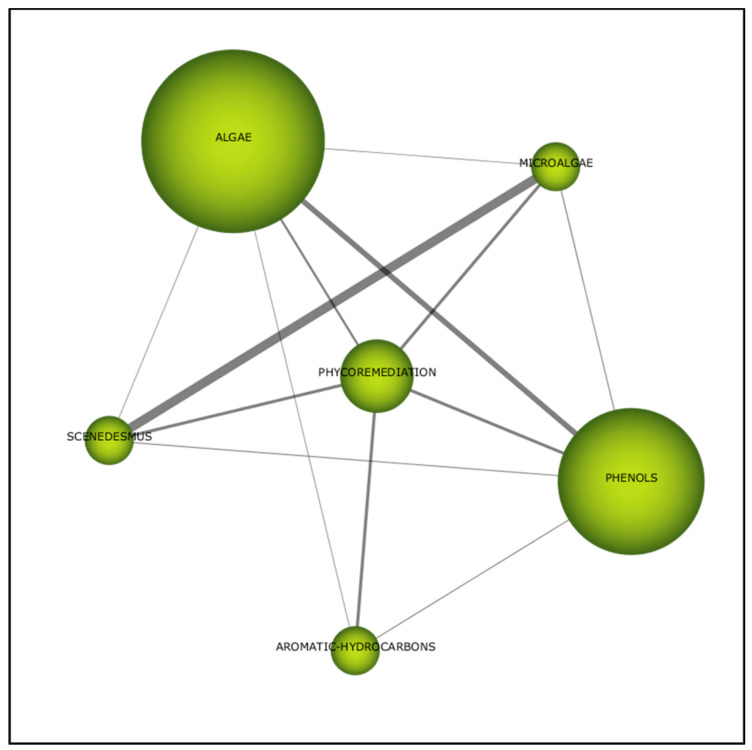
Thematic network for the first period (2000–2005).

“Phenol” was a component of the “phycoremediation” cluster in the first period (Figure 11). “Phycoremediation” is also highly related to “aromatic hydrocarbon” with the line weight value of 0.33 (Table 5). Besides, there are also connections formed among the sub-themes within the cluster. For instance, the term “phenols” is also highly linked to “algae” (Figure 11). Therefore, phycoremediation have been associated with features relating to phenol.

#### 3.2.2. Second Period (2006–2010)

**Figure 12:**
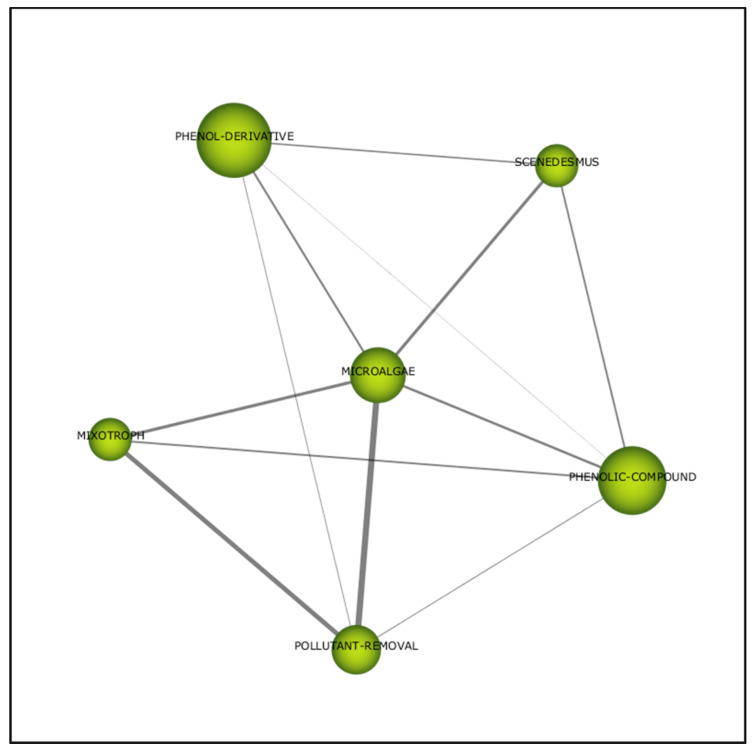
Thematic network for the second period (2006–2010).

In this second period, “microalgae” is the central cluster associated with five other themes (Figure 12). A high correlation (0.67) between “microalgae” and “pollutant removal” demonstrated the employment of microalgae in eliminating pollutant (Table 6). Mixotrophic algae can also be utilised to eliminate pollutants, since both themes are highly correlated. Hence, microalgae do exhibit an ability in removing pollutants, particularly phenolic compounds.

#### 3.2.3. Third Period (2011–2015)

**Figure 13:**
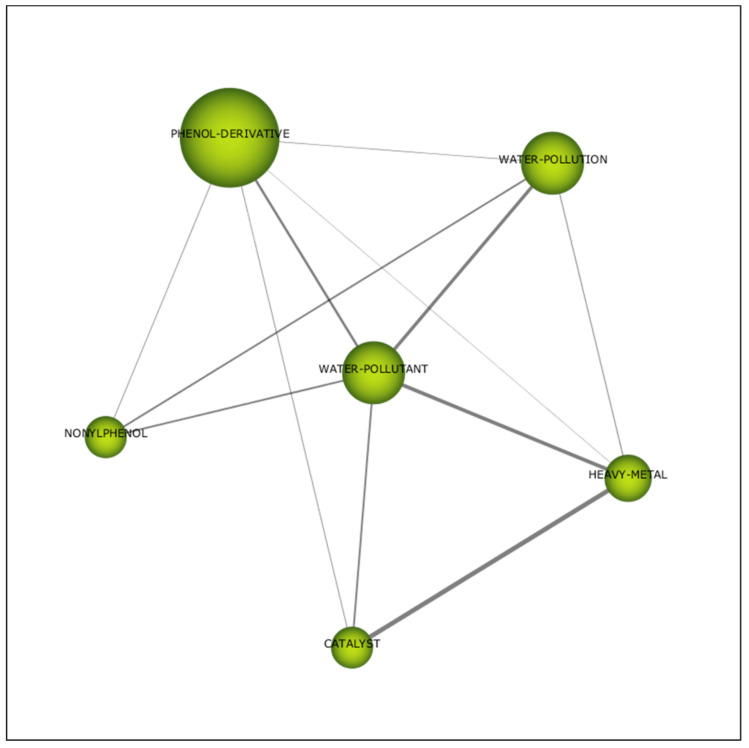
Thematic network for the third period (2011–2015).

The interrelation of all the themes concerned with water pollutants is illustrated in Figure 13. “Phenol derivatives”, “heavy metal” and “nonylphenol” are significant constituent elements of water pollutant. The network of topics connected to the central theme contains a diverse range of subjects that remain a significant link between them. “Water pollution” (0.67) was the relevant issue associated with water pollutant (Table 7).

#### 3.2.4. Fourth Period (2016–2020)

**Figure 14:**
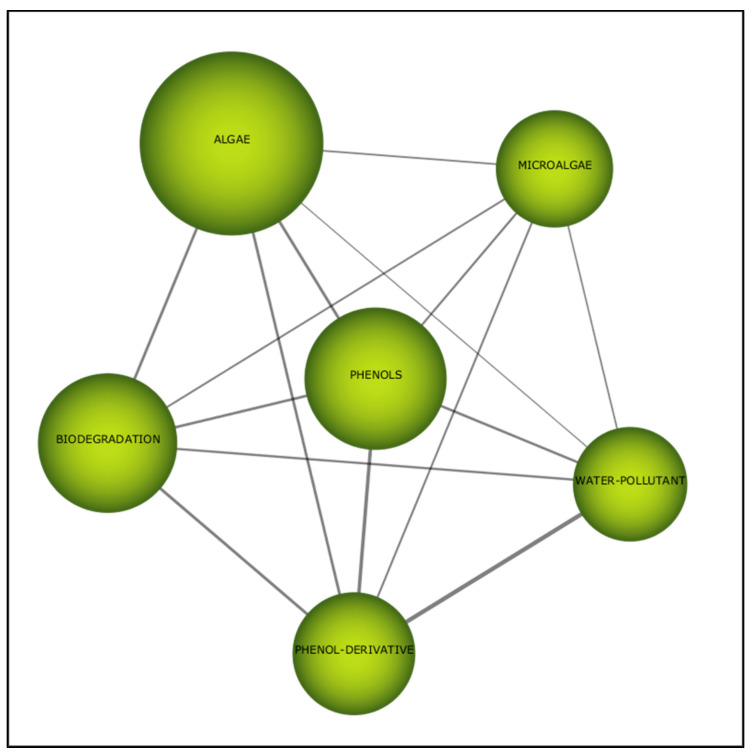
Thematic network for the fourth period (2016–2020).

The thematic network in this period provides a fascinating insight. “Phenols”, which is the central theme, is inextricably linked to sub themes “algae” (0.31), “biodegradation” (0.28) and “water pollutant” (0.27) (Table 8). This bolsters the efficacy of biodegradation by algae in research related to remediation of phenol. In the case of “microalgae”, despite the lack of strong correlation with the main cluster, the theme is still related to the issues of phenols (Figure 14). Therefore, algae showed the capability to biodegrade phenols at contaminated sites.

### 3.3. Evolution Map

**Figure 15:**
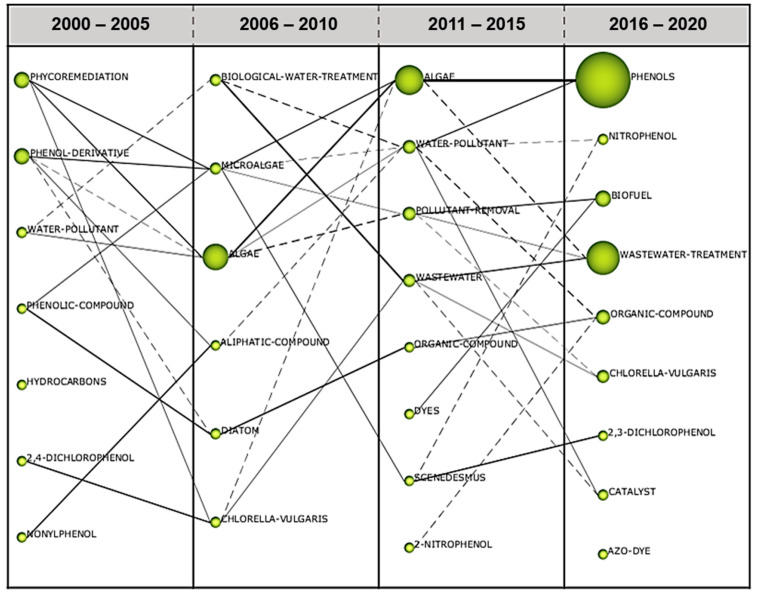
The evolution of thematic areas for the period of two decades (2000–2020).

The evolution map allows the analysis of conceptual evolution and, hence, adding weight to the argument in certain fields of study. This map is characterised by the size of the sphere and the thickness of the line. The sphere quantifies the number of publications, while the thickness shows the correlation between the themes of selected time frames [56]. From the year 2000 to 2005 and 2006 to 2010, there was a significant link between the term “phenol derivatives” and “microalgae” (Figure 15). This proved that the utilisation of algae in degrading phenolic compounds gained momentum in 2006. A strong liaison between the term “algae” and “phenols” can be seen between the period of 2011–2015 and 2016–2020 (Figure 15). The evolutionary path of algae has progressively evolved from a latent to growing state, hence, implying this research subject has a continued vitality in the phenol removal studies. 

## 4. Visualisation Using VOSviewer-Keywords Visualisation

VOSviewer emphasises the graphical representation of the map and facilitates exploring trends through keywords [57]. Principally, network data are exploited to construct the map. Through network analysis strategies such as co-citation, co-occurrence term and coupling, significant emphasis areas are pinpointed, resulting in discovering notable authors, publications, and journals [55]. VOSviewer is beneficial to visualise an outsized map that eases the interpretation as the distance between two terms often explains the relatedness of the terms [58].

**Figure 16:**
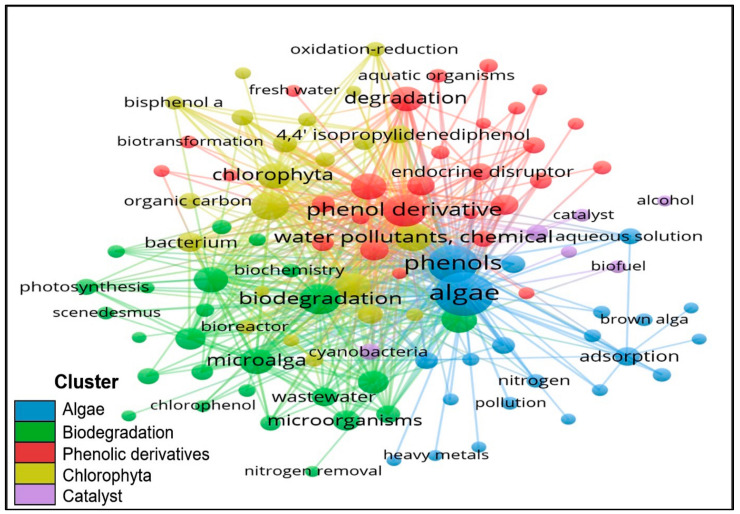
The network visualisation map of co-occurrence term.

Co-occurrence term analysis enables the search of limitations and hot trends in a certain topic. In this analysis, the cluster formed through the term that co-occurred frequently and the connection’s strength can be visualised by the thickness of the lines. In addition, the size of nodes indicates the number of keywords used, where the larger the size, the greater the registration number. The largest cluster (blue) with 28 items was closely related to algae (Figure 16). The term “algae”, which resided at the core of the map, was noted for its attention and linkage with other terms, as it featured a higher value in co-occurrence and total strength (Table 9). The cluster in green (25 items) focused on remediation approaches. The linkage of the term “biodegradation” and “algae” indicated that biodegradation has a significant contribution in removing pollutants by algae. 

### 4.1. Phenols 

Phenol is naturally found in coal tar and was first isolated in 1841 by Ferdinand Runge, a German Scientist [59]. It is one of the leading industrial discharges produced by manufacturing industries such as oil refineries, dye, pesticides, plastic plants and pharmaceutical industries. Phenol (C_6_H_5_OH) is the simplest member of phenolic compounds. Phenol and its derivatives are organic compounds comprising a hydroxyl group (-OH) bonded to one or more aromatic rings. Phenol was also notable as carbolic acid, benzophenol, or hydroxybenzene [60]. Chlorophenol, nitrophenol, methyl phenols, alkylphenols, aminophenols, butylhydroxytoluene, nonylphenol and bisphenols A are some other phenolic compounds.

#### 4.1.1. Sources of Phenol 

The production of phenol is done either naturally or synthetically with chemical processes. About 95% of the global synthetic phenol production is contributed by cumene oxidation [61]. The prime sources of phenolic waste are petroleum refineries, petrochemical, steel mills, coke oven plants, coal gas, synthetic resin, pharmaceutical, paints, plywood industries, mine discharge, explosive, the production of rubber goods, the textile industry as well as food and beverage [62,63,64,65,66]. Table 10 shows the details on the sources of phenol.

#### 4.1.2. Toxicity

The entry of the phenolic compound into the water bodies is due to the discharge of industrial waste. Ingestion of phenol (1 g) is detrimental to life [77]. In addition, phenolic compounds exhibit a foul odour and flavour in drinking water in relatively low concentration (5 μg/L). Numerous researchers have found phenol in industrial wastes at concentration ranging from 50 to 10,000 mg/L [78,79]. Besides, the dilution of phenol is slow, as it is heavier than water and leads to toxic compound formation even after being diluted. The concentration of phenol in seawater is generally low, with a concentration of only 0.13 mg/L even in polluted fishing areas. However, the phenol concentration has been recorded to rise up to 8.28/100 mL in the event of inadvertent spillage containing phenol into the sea owing to its high-water solubility [80].

Bisphenol A (BPA) is part of phenolic compounds. BPA is a plasticiser chemical used in polycarbonate polymers, plastics fabrication and epoxy resin [81,82]. BPA is resistant to biodegradation, although presented only at ppt level in the water [83].

Phenol is considered a safe disinfectant (concentration of 1% to 2% aqueous) and is used to treat non-critical medical devices with the lowest risk of infection transmission. Nevertheless, phenol is a dangerous pollutant that damages cells prolonged at concentration of 5 mg/L, and exposure to this disinfectant may counter skin irritation [77]. Toxicity limits for both human and aquatic life are typically within the range of 9 to 25 mg/L [84]. The wastes cause antibiotic-resistant genes in microorganisms, which concern public health [85]. Phenols are mostly volatile and release unpleasant odours in water, harming the aquatic organisms, interfere with the endocrine systems, destroy oxidative phosphorylation reaction, inhibit ATP production and accumulate in different trophic levels through the biological chain [1,51,86]. Humans absorb the phenolic compounds via inhalation, ingestion and skin contacts. Generally, the accumulation of phenol occurs in the brain, liver, muscle and kidneys [87]. Phenol is a protoplasmic poison that denatures proteins. The major organs damaged by phenol include the spleen, kidneys and pancreas [88,89]. According to Hansch et al. [90], two primary processes associated with phenol toxicity are (a) generation of free radicals and (b) non-specified toxicity linked to the hydrophobicity of each compound. Nitrophenol, chlorophenol and alkylphenols are relatively highly toxic [91]. The high distribution of phenols in nature implies widespread contact with humans and animals (Table 11). Phenol harms humans and animals, thus requires elimination to free the environment from contaminants. 

### 4.2. Algae

Algae are photosynthetic organisms that have shown high biological diversity and metabolic elasticity. They have better adaptability owing to their biochemical metabolic pathway and cellular composition responding to external conditions rather than terrestrial plants [13]. Algae are rich in biologically active compounds in macromolecules (proteins, fats, oils, and carbohydrates), antioxidants (polyphenol, tocopherol) and pigments [130]. Algae act as the primary producers in the biosphere as they are photoautotrophic microorganisms. Algae can be categorised into two types, which are microalgae and macroalgae [131,132].

**Figure 17:**
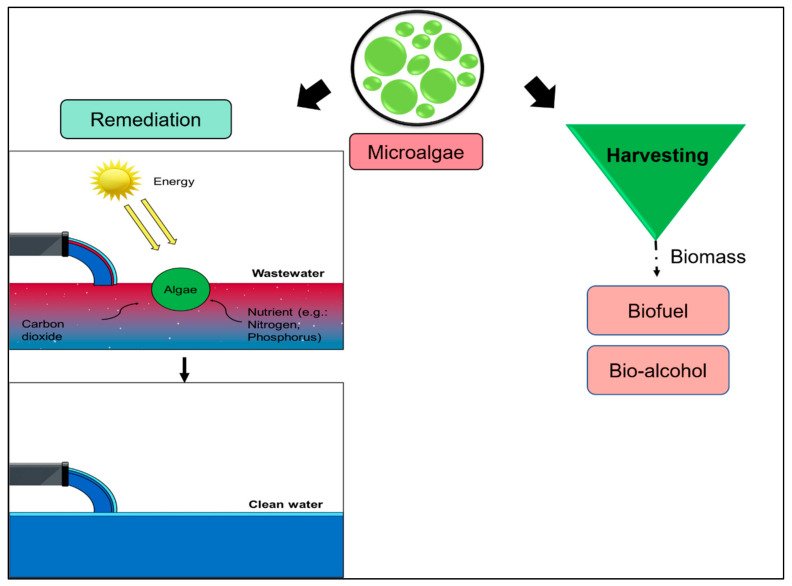
Cultivation of microalgae in treating wastewater.

Microalgae are the microscopic photosynthetic organism with a low-doubling time, which are comprehensively used in bioremediation, biodegradation and biofuel production (Figure 17) [133,134,135,136]. In recent years, biofuel has attracted substantial attention as a possible alternative energy source. Microalgae offers a great potential as a source of biofuel, as they develop rapidly and have great photosynthetic efficiency [137]. Additionally, microalgae are said to produce 10–100 times more fuel per unit area, unlike other sources like oil palm [138]. Therefore, microalgae are a promising alternative for the production of biofuel and reduce the reliance on fossil fuel that escalate the greenhouse gas emission.

Green microalgae with versatile metabolic networks can flourish in unfavourable conditions. Hence, green microalgae grown successfully in municipal, agricultural and industrial effluent reduce the micronutrient, nitrogen, organic and phosphorus content [139]. Interestingly, microalgae able to generate biomass by consuming the wastewater nutrient for high productiveness of biomass and value-added product [140]. When it comes to algal biomass, wastewater is the best resource according to multiple factors, such as it acts as a low-cost media and the availability of nutrients [141]. Microalgae are widely distributed in the aquatic environment and play a role as nutrient cyclers in the ecosystem. 

**Figure 18:**
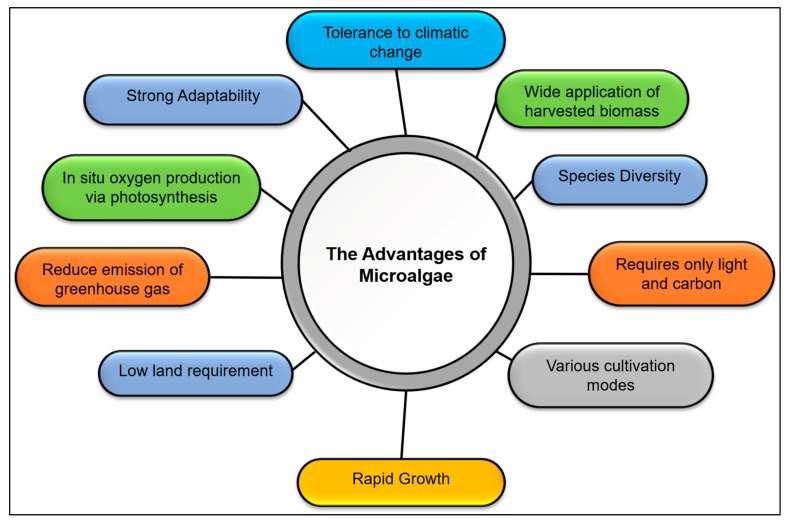
The benefits of microalgae.

The use of microalgae is especially beneficial in treating contaminants due to several reasons (Figure 18). Microalgae possess wide application due to their high biodiversity, genetic and metabolic engineering progress, and the growth of screening techniques [142]. *Chlorella* and *Scenedesmus* are well notable among others in eliminating wastewater contaminants. *Chlorella pyrenoidosa* and *Scenedesmus obliquus* both are capable in removing progesterone and norgestrel found in wastewater [143]. Besides, *Chlorella vulgaris* able to draw out dyes and heavy metal such as chromium, lead and molybdenum [144,145,146]. Hence, microalgae can be applied in treating wastewater from pharmaceutical, textile and beverage industries [147,148]. 

Furthermore, microalgae have high growth rates and the ability to fix carbon dioxide. The efficiency of carbon dioxide fixation by microalgae is 10–15% higher than terrestrial plants [149,150]. Thus, reducing industrial-scale carbon footprint [151,152]. In environmental biotechnology, microalgae are enriched toward biotransformation processes such as biodegradation owing to their specific metabolism [153]. Microalgae can be cultivated using wastewater and waste rich in organic and inorganic nutrients [154,155]. Water is also necessary for microalgal growth, as it regulates the temperature and provides a medium for nutrient delivery [156]. In addition, microalgae can also be utilised as a biocatalyst that further enhances the ecosystem’s protection against organic pollutants and hazardous metal ions [157,158]. Thus, microalgae are the potential candidates for the bioremediation of many pollutants. 

Microalgae are highly adaptable, in that they can thrive autotrophically, heterotrophically and mixotrophically [52]. The most common cultivation modes of microalgae are photoautotrophic and heterotrophic [159]. Photoautotrophic and heterotrophic processes are beneficial for biomass production and bioremediation. The mixotrophic condition exploits the advantages of both modes to conquer the disadvantages [160]. Light and organic carbon are not the limiting factors for the growth of microalgae in a mixotrophic condition. Mixotrophic microalgae can be utilised as distinctive agents for organic pollutant degradation. They can react to several organic pollutants in different ways, from biosorption to biodegradation [52]; therefore, becoming a potential candidate for phycoremediation of phenol. 

Although microalgae confer multiple obvious advantages, there are also cons linked with them. Microalgae can generate toxic compounds in wastewater as they generate oxygen to degrade phenol, polycyclic aromatic hydrocarbon and organic solvents [161,162]. Besides, the process is tedious than other approaches. Variability in light intensity and temperature over the course of the year may also hamper the growth of microalgae since they need sunlight to grow. Additionally, an adaptation of strains of microalgae on contaminated sites is needed, as sudden exposure to wastewater with very high contaminants is harmful to the culture [12].

### 4.3. Phenol-Degrading Algae

The accentuation of phenol degradation by algae has led to isolation, culture adaptation and enrichment that can thrive solely on phenols as a carbon and energy source [163]. As an antimicrobial agent, phenol is vulnerable to many microorganisms. Nevertheless, some phenol-resistant microalgae can degrade phenol (Table 12). 

Phenol and its derivatives are growth inhibitors for many green microalgae and require a lot of energy to be degraded. Kahru et al. [164] reported that phenol is harmful even to microalgae at as low as 0.05% concentration. Microalgal bioremediation has the ability for simultaneous carbon dioxide fixation via photosynthesis and contaminants degradation. Phototrophic and heterotrophic microalgae are sensitive to phenolic derivatives, yet mixotrophic microalgae can mineralise phenolic compounds [165]. 

Semple and Cain [166] stated that eukaryotic microalgae could degrade aromatic compounds such as phenol (Table 12). *Chlorella* and *Scenedesmus* are among the several strains commonly used to biodegrade phenolic compounds [167,168]. These strains can biodegrade a spectrum of phenolic compounds; for instance, 4-nitrophenol, 4-chlorophenol, 2,4-dinitrophenol and bisphenol [169,170,171,172], nonylphenol [173], pentachlorophenol [165] and 2,4-dimethylphenol [163,174]. *Scenedesmus obliquus*, *Chlorella* sp. and *Spirulina maxima* were the first three strains reported to degrade phenols in cultures [170]. Later, *Ochromonas danica* showed the ability to grow heterotrophically with phenol, where *p*-cresol acts as its sole carbon substrate [166]. 

A study conducted by Nazos et al. [175] stated that, in *Chlamydomonas* cells, phenol is only biodegraded when the algae need carbon reserves to maintain homeostasis. The versatile bioenergetic machine of *Chlamydomonas reinhardtii* regulates its metabolism to ensure a good balance between growth and biodegradation of phenol [175]. The availability of mechanical insight propounds the employment of marine and freshwater microalgae for phenol biodegradation. Therefore, microalgae are thought to be efficient in the removal of hydrophobic organic pollutants.

### 4.4. Insight into Biodegradation

The microalgal biodegradation process proceeds either intracellularly or extracellularly, or a combination of both. The initial degradation is done extracellularly and further degradation is carried out intracellularly [191,192]. The bio-uptake of contaminants by the cells involves intracellular degradation, while extracellular degradation is dependent on the excretion of enzymes that function as an external digestive system. However, the significant drawbacks of biodegradation are the challenge to control the optimal level of growth media, not suitable for a high concentration of phenol (greater than 2.5 g/L) and may require co-solvent such as ethanol when the phenol concentration is low [193,194,195].

#### 4.4.1. Factors Affecting Phenol Degradation by Algae

It is essential to understand the contribution of factors affecting the microbe’s degradation profile as biodegradability depends on several factors. Choosing a suitable physiological condition is always a key challenge as traditional experimental design necessitates numerous experimental runs to acquire a decent outcome. Alternative carbon sources, light intensity, phenol concentration, initial algal concentration, oxygen availability and temperature are a set of factors affecting phenol degradation [196,197]. Microalgal cells require an alternative carbon source and sufficient light intensity to biodegrade phenols. Furthermore, the addition of alternative organic carbon sources lowers the toxicity of phenolic compounds and promotes algae development [51,198]. At the same time, alternative carbon sources help to reduce the stress response induce by phenol toxicity [198]. However, in the unavailability of acetic acid, *Chlamydomonas* cells uptake phenol more readily in the first 48 h of incubation, since phenol is the only carbon source in the medium that causes the cell to generate carbon reserves to meet their carbon needs for homeostasis and cellular structure [175]. Similarly, the microalgae strain of *Chlorella fusca* var. vacuolata and *Anabaena variabilis* degrade phenolic compounds without organic carbon sources [169]. 

Exogenous glucose had been shown to improve halophenol degradation. On the contrary, Lika and Papadakis [199] reported that glucose slows down the phenol degradation due to the competition for oxygen by the heterotrophic absorption and phenol degradation. Hence, the availability of alternative carbon that stimulates microalgae development may limit biodegradation, since the substrates require enough oxygen to be metabolised.

The presence of phenol in the cultivation of marine microalgae upregulates genes attributable to reactive oxygen species (ROS) production and chlorophyll content reduction [169]. Moreover, the biodegradability of *Scenedesmus obliquus* on various forms of monosubstituted phenols is reliant on the culture condition used and the types of phenolic compounds studied [200]. Comparatively, acetic acid inhibits microalgal growth compared to cultures grown with the absence of phenol in the tris-acetate-phosphate (TAP) medium.

In response to stress, higher concentrations of phenol induce higher biodegradation levels in *Chlamydomonas reinhardtii*. Conversely, lower concentration of phenols and monosubstituted methylphenol, with the exclusion of alternative carbon sources in the culture medium, increased biodegradability [197]. In the case of *Cyclotella caspia*, the elevated concentration of nonylphenol reduced chlorophyll content and cell growth rate [201]. A low concentration of phenol is not harmful to microalgae but acts as a potential carbon source. However, high phenol levels inhibit algal growth as phenol induced phenoxy radicals causes damage to the membrane bound cellular organelles and photosynthetic pigment [202]. Hence, high concentration of phenol substantially restricts the algal growth. 

Light is also a pivotal factor in phenol degradation of microalgae. The degradation of phenolic compounds decreases under high light intensities cause by increased toxicity from the autoxidation process enhanced by light [203]. According to Wurster et al. [183], phenol was only biodegraded in the dark and not in the photoautotrophic and photoheterotrophic conditions as observed in *Synechococcus* PCC 7002. Similarly, *Scenedesmus* sp. performed better in heterotrophic than mixotrophic condition. This result may be explained by the fact that there is a decrease in light penetration in the mixotrophic system, suggesting the critical role of light in phenol degradation [204]. On the contrary, *Isochrysis galbana* requires light intensity of 180 μmol m^−2^s^−1^ to completely remove phenol, concentration of 50 and 100 mg/L within 14 and 24 h respectively [205]. Additionally, living *Chlorella* sp. also degrade phenol effectively under light condition while there is no significant biodegradation takes place under dark condition. Interestingly, the *Chlorella* cell began to degrade phenol after being exposed to light [163]. Besides, a mathematical model showed that phenol degradation was improved by increasing the light intensity due to the increase of photosynthetic oxygen production [199]. It further shows that incorporating inorganic carbon sources such as carbon dioxide and sodium bicarbonate can enhance both microalgal growth and biodegradation rate under increased light intensities. The shortage of oxygen may be the limiting factor during the peak phase of phenol biodegradation. 

Temperature is indeed one of the parameters affecting the biodegradation of phenol by microorganism. As mentioned by Li et al. [205], a lower temperature (10°C) hampering the removal procedure by *Isochrysis galbana*. This is due to the inhibition of enzyme which retards their growth and metabolism. A higher temperature enhances the activity of photosynthesis-related enzymes, as well as key processes such as carbon dioxide diffusion [206]. Higher temperature hastens the process of cellular metabolism, thereby promoting microalgal growth. However, there will be an irreversible physiological reaction taking place in the cell as the temperature exceeding the optimum temperature. Thereby, impacting the growth rate and photosynthetic rate of algae.

#### 4.4.2. Elucidation of Mechanism and Enzymatic Action on Phenol Degradation

The phenol degradation by algae especially microalgae proceed aerobically [207]. Aerobic microalgae metabolise aromatic compounds since they can adapt to unfavourable conditions. The cleavage pathways vary among microalgal species. Hence, the study on the enzymatic reactions, particularly the degradation and detoxification of phenol, had drawn many researchers’ consideration. Photosynthetic and metabolic activities influence the biodegradation ability of microalgae. The photosynthetic nature of microalgae allows the generation of toxic oxygen species that act as strong oxidising agents such as O_2_^−^, OH^−^ and H_2_O_2_. Molecular enzymes are necessary to initiate the enzymatic attack on the aromatic phenol rings [166,208]. On that account, microalgae require molecular oxygen for the enzymatic breakdown of phenol. 

Phenol-degrading enzymes, such as lignin peroxidase, laccase, polyphenol oxidase, superoxide dismutase, catalase, peroxidase and ascorbate peroxidase, occur in many species of microalgae. Cytochrome P450 is also involved in the phenolic compound biodegradation by *Chlorella* sp. [207]. Microalgae can secrete extracellular polymeric substances (EPS), protein and numerous types of hydrocarbons similar to bacteria. The EPS serve as surfactants and emulsifiers to improve the bioavailability of contaminants for subsequent cell uptake [209]. Enzymes are crucial in biodegradation by increasing the hydrophilicity of the pollutant. This can be accomplished by adding a hydroxyl group via hydrolysis, oxidation, or reduction [209]. Phenol hydroxylase is involved in the hydroxylation of phenol to catechol, in which the enzyme catalyses the attachment of the hydroxyl group to the *ortho*- position of the aromatic ring. Hydrogen donor reduces the other oxygen atom to water. Phenol hydroxylase also catalyses the hydroxylation of hydroxyl-, amino- or methyl-substituted phenol besides phenol [210], which is generated by strong oxidative products of the reaction, catechol [16,211]. Interestingly, catechol can also be hydroxylated by phenol hydroxylase into pyrogallol. The formation of pyrogallol can be observed at high substrate concentrations as phenol is the only substrate for the enzymatic reactions [16,211]. 

Under the aerobic condition, degradation of phenol is initiated by oxygenation, with aromatic rings initially monohydroxylated to catechol by a monooxygenase phenol hydroxylase at an *ortho*- position to a pre-existing group [16]. All monooxygenases incorporate one atom of oxygen in the respective substrate. Catechol is the primary intermediate formed when various strains metabolise phenol. Numerous similarities can be drawn between pathways discovered in bacteria and unicellular microalgae. Later, the cleavage of catechol proceeds either at *meta*- or *ortho*-position. The activity of the enzymes differentiates both pathways. 

**Figure 19:**
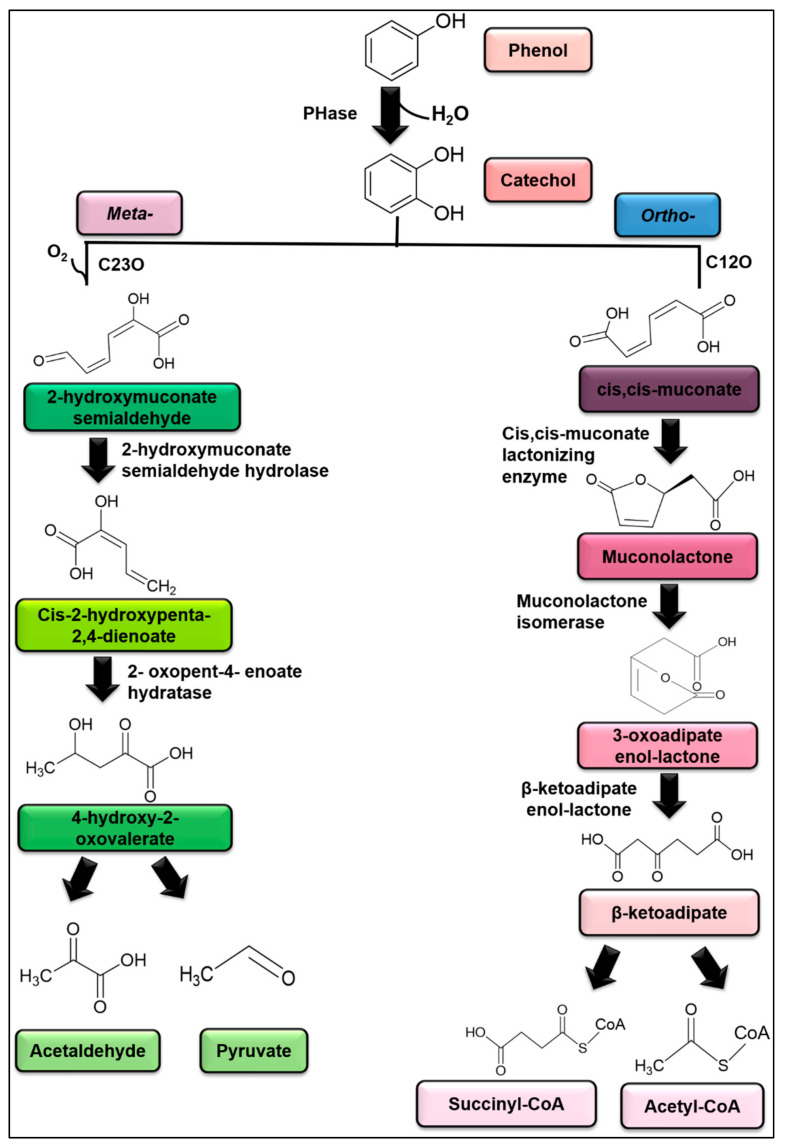
The proposed phenol degradation pathway by Das et al. [177]. PHase: Phenol hydroxylase; C12O: Catechol-1,2- dioxygenase; C23O: Catechol-2,3-dioxygenase.

The *ortho*-pathway is initiated when the ring of catechol is cleaved at the *ortho*- position. This pathway is facilitated by the 1,2-dioxygenase enzyme consisting of a prosthetic group of Fe^3^+, which leaves two carbons connected with the hydroxide group into cis,cis-muconic acid [175,212]. Succinyl-CoA and acetyl-CoA are formed from the intermediates following a series of steps (Figure 19). Microalgae can extracellularly undergo *ortho-* reaction with the phenolic compound in the dark. 

Besides, the *ortho*-pathway also predominates the phenol metabolism of *Isochrysis galbana* than the *meta*-cleavage [180]. The study reported that catechol 2,3-dioxygenase actively participated in the cleavage of the benzene ring of the *ortho*-position. Catechol-1,2-oxygenase was also noted exhibiting activity higher than catechol-2,3-dioxygenase in *C. pyrenoidosa* (NCIM 2738). This demonstrated the *ortho*-pathway of benzene rings. On the other hand, Das et al. [177] reported that *C. pyrenoidosa* was able to biodegrade phenol through both *ortho*- and *meta*-pathways. However, the *ortho*-pathway was more dominant due to the accumulation of catechol, cis, cis-muconic acid and 2,3-hydroxy-muconic semialdehyde intermediates in the growth medium. 

Following *meta*-pathway, 2,3-dioxygenase occupying with Fe^2+^ prosthetic group cleaves adjacent carbon-carbon bonds of one hydroxide group results in 2-hyroxymuconic semialdehyde [212]. The intermediate is further metabolised into pyruvate and acetaldehyde (Figure 19). *O. danica* possesses *meta*-cleavage of phenol and its methylated homologues enzymatically. Pyruvate is formed due to prolonged incubation of muconic semialdehyde with the cell-free extract [208]. Therefore, ring cleavage can occur in two orientations.

A high concentration of phenol can inhibit the activity of phenol hydroxylase. Wang et al. [180] reported that the intracellular enzymes mainly catalysed the phenol in *Isochrysis galbana*. They discovered that the high concentration of phenol inhibited the activity of phenol hydroxylase; however, no effect was observed on catechol dioxygenase. The inhibition of biodegradation of high concentrations of phenol by microalgae might be due to the inhibition of phenol hydroxylase. The activity of phenol hydroxylase under high phenol concentration can be improved by long term phenol acclimation or through genetic modification of the microalgal strain. In addition, the toxicity of phenol to microalgae can be reduced through the presence of organic carbon sources [198]. Polyphenol oxidase and laccase, which are inducible intracellular enzymes, are also involved in the phenol metabolism of algae. Hence, the sensitivity of microalgae to phenolics compounds can be explained to be due to the number and polarity of aromatic ring substituents.

## 5. Conclusions

This review sought to assess the publishing patterns in the research of phenol degradation by microalgae for the period of 2000–2020 based on the Scopus database. Bibliometrics aids in the development of future research and assists researchers in identifying interest in respective fields of study. In terms of publication trends, studies on phenol degradation by microalgae shows a fluctuating trend, suggesting that this topic is a developing research topic. 

Phenolic compounds need to be removed to protect the environment. Biological treatment is environmentally sustainable, cost-effective and the most effective technique available. This treatment has gained growing interest in pollution control. Algae are an essential part of natural ecosystems that mediate the biodegradation of phenol. They can thrive in a harsh environment beneficial for rapid and efficient removal of phenolic contaminants. The biodegradation involves the breakdown of an organic compound into compound with less complexity via biotransformation. Algae metabolism is an energy transfer process regulated by enzymatic processes, where intermediate reactions play an essential role. Biodegradation is a versatile process that includes several important factors. The degradation of phenol and its derivatives by algae has been the focus of scientific interest for many decades. Microalgae biodegrade many natural and synthetic organic compounds as part of their regular energy and growth metabolism. Organic material that acts as a primary electron and energy source is converted to oxidised end products via redox reactions. The other part of the organic carbon is synthesised into cellular materials. This conversion proceeds in an aerobic environment with oxygen as the terminal electron acceptor. The action of enzymes involved in aromatic catabolism is crucial for developing more effective and modern treatment technologies. Hence, research in the specificity of phenol biodegradation by algae, especially microalgae, is essential for developing useful remediation approaches.

## Figures and Tables

**Table 1:** The measures for themes of the first period (2000–2005).

Cluster	h-Index	Centrality	Density
Phycoremediation	2	0.50	1
Phenol derivatives	2	0.83	0.83
Water Pollutant	9	1	0.33
Phenolic compound	1	0.14	0.43
Hydrocarbon	1	0.29	0.57
2,4-dichlorophenol	1	0.57	0.71
Nonylphenol	1	0.43	0.14

**Table 2:** The measure for themes of the second period (2006–2010).

Cluster	h-Index	Centrality	Density
Biological water treatment	3	0.67	1
Microalgae	3	0.83	0.67
Algae	17	1	0.50
Aliphatic compound	1	0.17	0.83
Diatom	2	0.33	0.33
*Chlorella vulgaris*	2	0.50	0.17

**Table 3:** The measures for themes of the third period (2011–2015).

Cluster	h-Index	Centrality	Density
Algae	18	1	1
Water Pollutant	4	0.62	0.88
Pollutant removal	5	0.88	0.75
Wastewater	4	0.75	0.25
Organic compound	1	0.25	0.50
Dyes	1	0.12	0.62
*Scenedesmus*	1	0.50	0.38
2-nitrophenol	1	0.38	0.12

**Table 4:** The measures for themes of the fourth period (2016–2020).

Cluster	h-Index	Centrality	Density
Phenols	19	1	0.89
Nitrophenol	1	0.56	1
Biofuel	6	0.67	0.44
Wastewater treatment	11	0.89	0.67
Organic compound	5	0.78	0.33
*Chlorella vulgaris*	4	0.44	0.22
2,3-dinitrophenol	1	0.22	0.56
Catalyst	1	0.33	0.11
Azo dye	1	0.11	0.78

**Table 5:** The weight of lines connected to main theme “phycoremediation”.

Member	Weight
Phenols	0.33
Aromatic hydrocarbon	0.33
*Scenedesmus*	0.33
Algae	0.25
Microalgae	0.33

**Table 6:** The weight of lines connected to the main theme “microalgae”.

Member	Weight
Phenolic compound	0.27
Pollutant removal	0.67
Mixotroph	0.33
Phenol derivative	0.22
*Scenedesmus*	0.33

**Table 7:** The weight of lines connected to the main theme “water pollutant”.

Member	Weight
Heavy metal	0.27
Catalyst	0.10
Water pollution	0.36
Nonylphenol	0.17
Phenol derivative	0.22

**Table 8:** The weight of lines connected to the main theme “phenols”.

Member	Weight
Algae	0.31
Water pollutant	0.27
Phenol derivatives	0.37
Biodegradation	0.28
Microalgae	0.23

**Table 9:** The number of co-occurrence and total strength of the research topic.

Keyword	Occurrence	Total Link Strength
Algae	116	1008
Phenols	79	810
Phenol derivatives	59	705
Biodegradation	50	543
Water pollutant, chemical	42	574
Green alga	41	528
Phenolic compounds	37	447
Biomass	36	367
Microalga	35	436
Chlorophyta	35	443
Pollution removal	35	506
Bioremediation	34	421
Degradation	33	298
Enzyme activity	33	153
Wastewater treatment	27	337
Biodegradation, environment	25	328

**Table 10:** The sources and application of phenolic compounds in various industry.

Industry/Sources	Compound	Used in Application	References
Agriculture	Phenol and acetone	Production of pesticides, fungicides, and herbicides in such 2,4-dichlorophenoxyacetic acid.	[67]
	Monoisopropylamine products	Protection of crop and increase yield	
Automotive	Phenolic resins	Manufacture filters, tires, insulation, and coating additives	[68,69]
	Phenol	Generation of polycarbonate for automotive parts	[70]
	Nylon intermediates	Manufacture of thermoplastics and carpeting	
Construction	Phenolic resin	Concrete forming, insulation, beams, moulding compounds	[68]
	Bisphenol A	Plastic pipes	[71]
Cosmetic	Benzophenone-3	Sunscreen	[72]
	Phenol	Used in chemical skin peels, formulation of lip balm	[73]
Household	Phenol, Benzophenone-3	Manufacture of soaps, paints, toys, lacquers, and perfumes	[71,74]
Food and beverage	Bisphenol A	Coating of cans, cups, and polycarbonate container	[71]
Pharmaceutical	Phenol	Antiseptic, slimicide, lotion, ointment, mouthwash, oral spray for treating sore throat	[60]
Plywood	Pentachlorophenol	As wood preservatives	[75]
Textile	Caprolactam and adipic acid (Intermediate of phenol)	Production of synthetic yarn	[76]

**Table 11:** The toxicity of phenolic compounds.

Compounds	Organism	Effects	Details	References
Phenol	Human	Blister and burn on the skin	Coagulation is associated with phenol and amino acid reaction in the keratin of the epidermis and collagen	[60,92]
		Heart failure	Ingestion of high concentration of phenol (70 ml of 42–52% phenol)	[93,94,95]
		Acute renal failure	Exposure to 40% of phenol in dichloromenthane	[96]
		Necrosis	In contact with phenol solution (concentration of 1%)	[87,93,97,98]
		Dry mouth and throat, dark urine, and diarrhoea	Via ingestion of a high concentration (10–240 mg/L) of phenol	[87,96,99]
		DNA and chromosomal damage in leukaemia inhibit Topoisomerase and clonal selection process	Effect of benzene-related hematotoxicity	[100,101]
		Cause anorexia, weight loss, headache, muscle pain, jaundice	Chronic toxicity due to vaporisation of phenol	[102,103]
	Animal	Increase gill necrosis and mucus production	Interference with respiration	[104]
		Asphyxia		[105]
		Destruction of erythrocytes		
		Hypocholesterolaemia	Manifesting uptake of cholesterol in corticosteroidogenesis	[106]
		Modify aquatic biotas such as algae and other microorganisms	A high concentration of phenol is lethal	[107]
		Cause bronchoconstriction and adverse effects in rat	Low phenol concentration (0.1%) causes strong bronchoconstriction	[108]
		Toxicity to bone marrow	Generation of free radical and electrophilic intermediates during peroxidase-dependent oxidation	[109,110]
		Changes in skin, urogenital tracts, lungs and liver	Generated by lipid peroxidation which damages and eventually degrades the membrane of the cell	[87]
Catechol	Human	Acrylation	Due to the generation of hydrogen peroxide, superoxide, and hydroxyl radicals	[111,112]
		Destruction of a particular protein in the body	The reaction between catechol with sulphydryl groups of both protein and glutathione	[111]
		Disruption of electron transportation in energy-transducing membranes	Result of the tendency of phenol to oxidise quickly to quinone radical that is more reactive	[111]
		Lead to death	The dose of 50–500 mg/kg of body weight	[87]
Chlorophenol	Human	Burns of mouth and throat, white necrotic lesion in the mouth, stomach, and oesophagus	Acute poisoning	[113]
		Vomiting and headache		[114]
		Injury to the digestive tract, liver, kidney, lungs, and skin		[115]
		Hypotension and abdominal pain	Chronic toxicity	[87]
		Suppress immune system	Through drinking of water or eating food containing chlorophenol	[93,98,116]
		Hypothermia, pulse fluctuation, muscle weakness, and seizures	Exposure to concentrated phenol	[113]
	Animal	Disturb organ and endocrine system in aquatic organism	Disruption of free radical metabolism, the immune response factor	[117]
		Inhibit cell growth and induce genetic mutation in fish	Low concentration elevates point mutation on the zebrafish genome	
Hydroquinone	Human	Damaging chromosomes	Through the generation of reactive oxygen species (ROS)	[118,119,120,121]
Bisphenol A	Human	Alter development of the mammary gland	BPA is an oestrogen compound that can also interfere with androgen activity	[122,123]
		Delay onset of puberty among girls	Mimicking oestrogen action	[124,125]
		Metabolic disorder and abnormalities among babies	It is linked to a low dosage of BPA and estrogenic activity	[126,127,128]
		Cause breast and prostate gland cancer		[126,127,128]
	Animal	Cause mutation and retardation of the animal reproductive system	Accumulation of BPA in the environment	[129]
2,4-dimethylphenol	Human	Skin and eye irritation, asthma, anoxia, and eczemas	Due to the initiation of semiquinone and superoxide radicals, which harm the cell’s biomolecule	[102]

**Table 12:** The phenol-degrading algae.

Compound	Phenol-Degrading Algae	Efficiency	References
Phenol	*Ankistrodesmus braunii*	Removal of over 70% of phenol from olive oil mill wastewater within 5 days.	[176]
	*Chlorella* sp.	Degraded 1000 mg/L of phenol in less than 6 days. There is no rapid degradation observed at higher concentrations (3000 mg/L).	[170]
		Degrade 500–700 mg/L phenol within 7 days under continuous illumination.	[53]
	*Chlorella pyrenoidosa*	Degrade up to 60% of phenol at all concentration.	[32]
		Degrade with maximum phenol concentration of 200 mg/L under optimal condition.	[177]
	*Chlorella vulgaris*	Removed 98% at high phenol concentration (100 mg/L) after 4 days.	[178]
	*Chlorella* sp.-*Cupriavidus necator*	Could degrade phenol with the maximum concentration of 1200 mg/L within 60 h under optimal condition.	[179]
	*Isochrysis galbana* MACC/H59	Complete degrade phenol at the concentration of 100 mg/L within 4 days. It also degrades 50 mg/L phenol within 2 days. Lower concentration stimulates growth. The maximum concentration that can be degraded is 200 mg/L.	[180]
	*Phaeodactylum tricornutum* MACC/B114	Require 8 days to degrade 50 mg/L of phenol and 10 days for 100 mg/L.	[180]
	*Phormodium valderianum* BDU 30501	They were grown in 50 mg/L of phenol concentration and removal of 38 mg/L within 7 days retention period. Inhibition of the growth occurs at the concentration of 100 mg/L	[181]
	*Scenedesmus regularis*	Remove 40% of phenol. The optimal phenol concentration is 30 mg/L.	[182]
	*Scenedesmus quadricauda*	Resistant to phenol, they degrade low molecular weight phenol found in olive oil mills wastewater through biotransformation. High removal of monophenol (over 50%) in the dark.	[176]
	*Spirulina maxima*	Removed 97.5% of phenol at phenol concentration of 50 mg/L within 24 h.	[17]
		Degraded 1000 mg/L of phenol after the adaptation period.	[170]
	*Synechococcus* PCC 7002	Degrade phenol concentration of 100 mg/L in 5 to 7 days under a non-photosynthetic condition in the dark.	[183]
	*Tribonema minus*	Highest removal (94.6%) at the concentration of 250 mg/L.	[184]
2,4-dinitrophenol (2,4-DNP)	*Anabaena variabilis* NIES 23	Removed 86% 2,4-dinitrophenol with an initial concentration of 40 µM and cultivated for 72 h.	[169]
	*Chlorella* sp.	Degrade 70 mg/L of 2,4-DNP in 20 days.	[170]
	*Scenedesmus obliquus*	Degrade 190 mg/L of 2,4-DNP.	
Bisphenol A (BPA)	*Chlorella fusca* var *vaculota*	Able to remove most BPA in the range concentration of 10 to 80 µM for 168 h under continuous illumination.	[185]
	*Chlorella vulgaris*	Biodegrade 23% of BPA at the concentration of 1 mg/L BPA. Rapid degradation occurs at this concentration.	[186]
	*Chlamydomonas mexicana*	Degrade 24% of BPA at the concentration of 1 mg/L. Increasing the concentration of BPA caused an increase in carbohydrates levels in the cells due to the stress effect.	
	*Monoraphidium braunii*	Removed 48% of BPA at the concentration of 4 mg/L. The growth inhibited at high concentrations.	[187]
	*Stephanodiscus hantzschii*	Removed 99% of BPA in media supplemented with 0.10 mg/L BPA after 16 days of treatment. The biodegradation activity decreases with increased BPA concentration. The algal growth and biodegradation activity inhibited at higher BPA concentrations. The cell reached the death phase earlier than the control.	[188]
Nonylphenol (NP)	*Ankistrodesmus acicularis*	Removal rate of 83.77% after 120 h of exposure to different NP concentration (0.5–2.5 mg/L).	[189]
	*Chlorella vulgaris*	Degraded over 80% of NP after 168 h.	[173]
	*Platymonas subcordiformis*	Removed 82.34% of NP of its initial concentration after 5 days of culture.	[190]
*p*-chlorophenol	*Chlorella vulgaris* and *Coenochloris pyrenoidosa* (Microalgal consortium)	Remove *p*-chlorophenol under different light regimes. Able to degrade 50 mg/L of *p-chlorophenol* under 24 h light regime within 5 days.	[172]

## Data Availability

Not applicable.
